# Human papillomavirus E7 oncoprotein expression by keratinocytes alters the cytotoxic mechanisms used by CD8 T cells

**DOI:** 10.18632/oncotarget.23210

**Published:** 2017-12-14

**Authors:** Purnima Bhat, Anne-Sophie Bergot, Nigel Waterhouse, Ian Hector Frazer

**Affiliations:** ^1^ University of Queensland Diamantina Institute, University of Queensland, Translational Research Institute, Brisbane, Qld, Australia; ^2^ Medical School, Australian National University, Canberra, Act, Australia; ^3^ QIMR Berghofer Medical Research Institute, Brisbane, Qld, Australia

**Keywords:** cervical cancer, head and neck cancer, immunotherapy, cell-mediated killing, time-lapse microscopy

## Abstract

Cervical cancer is a malignant transformation of keratinocytes initiated by the E7 oncoprotein of human papillomavirus (HPV). These tumors are characterized by keratinocyte hyperproliferation and are often infiltrated with activated CD8 T cells. HPV infection confers changes to gain immunological advantage to promote chronic infection, and these persist with malignant transformation. We investigated the relative importance of the many redundant mechanisms of cytotoxicity used by CD8 T cells to kill keratinocytes expressing HPV E7 oncoprotein using extended-duration time-lapse microscopy that allows examination of cell-to-cell interactions during killing. E7 expression by keratinocytes increased susceptibility to cell-mediated killing. However, while killing of non-transgenic keratinocytes was traditional, perforin-mediated, and caspase-dependent, E7-expression favored killing by perforin-independent, caspase-independent mechanisms. The roles of perforin, TNFα, IFNγ, Fas/FasL and PD1/PD-L1 were graded according to target cell survival to produce a hierarchy of killing mechanisms utilized in killing E7-expressing cells. TNFα was essential for perforin-mediated killing of E7-expressing cells, but not perforin-independent killing. IFNγ facilitated killing by Fas/FasL interaction, especially in the absence of perforin. Additionally, expression of E7 offered protection from killing by up regulation of PD-L1, Fas and FasL expression on keratinocytes promoting fight-back by target cells, resulting in effector cell death. This study shows that keratinocytes expressing E7 are highly susceptible to killing by CD8 T cells, but utilizing different armamentarium. Down-regulation of CD8 T cell cytotoxicity in HPV-related tumors may be due to suppression by E7-expressing keratinocytes. Immunotherapy for HPV-related cancers may be improved by suppression of PD-L1, or by suppression of FasL.

## INTRODUCTION

Persisting human papillomavirus infection is the main cause of cervical and anal cancers, and has been implicated in a large number of other epithelial tumors including skin, esophageal, and head and neck cancers [1]. Papillomavirus infects basal replicating keratinocytes (KC) and is cleared by the immune system in the majority of cases resulting in no further clinical progression. In persistent infection, HPV evades clearance by the immune system, increasing the risk for oncogenesis [2]. HPV proteins E6 and E7 are expressed in all cervical cancers, and are responsible for the oncogenic progression of lesions by their suppressive action on the retinoblastoma gene product, resulting in KC proliferation and suppression of differentiation. HPV infection results in maintaining cell cycling, preventing apoptosis, and preventing differentiation, due to expression of its two major oncoproteins, E6 and E7 [3].

Although the immune system has failed to detect and destroy these cells producing non-self, virus-derived, oncogenic proteins, these lesions are often associated with a marked immune cell infiltrate. The presence of CD8 T cells in cervical lesions is associated with a favorable prognosis, with their numbers inversely correlating with tumor progression [4, 5]. Despite infiltrating tumors, however, effector T cells are unable to kill their targets [6]. E7-expressing murine skin grafts placed on E7-naïve animals, are not rejected [7]. As skin graft rejection is CD8 T cell dependent, tolerance of E7-expressing skin suggests that the E7 oncoprotein plays a role in inhibiting cell-mediated immune function in cervical cancers.

Persistence and transformation of the basal KC by proteins such as E7 is associated with immune evasion. Multiple mechanisms of immune evasion within transformed KC have been described including Fas down-regulation [8, 9], downstream effects on apoptosis of down-regulation of the retinoblastoma gene [10], alteration of the processing of IFNγ -dependent intracellular proteins [11, 12, 13], and changes to the TNF receptor and processing [14]. Additionally, the effects of HPV on infiltrating lymphocytes, preventing activation [15], affecting mechanisms of cytotoxicity, or promoting anergy or exhaustion [15, 16] may be due to the expression of E7 oncoprotein.

CD8 T cells have a substantial armamentarium with which to kill target cells. Contact-dependent mechanisms including perforin/granzyme and Fas-FasL, and contact-independent mechanisms such as production of TNFα or IFNγ – are utilized by CD8 T cells in tumor environments with varying degrees of preference.

We examined the mechanisms by which activated antigen-specific CD8 T cells may kill keratinocytes expressing E7 protein. Using a live-cell imaging-based killing assay, we co-cultured primary keratinocytes from mice expressing E7 from the keratin-14 promoter (E7KC), or from non-transgenic mice (B6KC), loaded with OVA, and EGFP^+^CD8^+^ effector T cells from OVA-immunized mice. These studies allowed us to examine the interactions between targets and effectors during the attachment and killing process and the kinetics of killing. E7KC were more susceptible to killing by CD8 T cells than their non-transgenic counterparts, however, it was apparent that effectors were using a different set of mechanisms to achieve target death. Additionally, E7KC themselves produced proteins such as FasL and PD-L1, lethal to cytotoxic T cells. Recently, utilizing an anti-CD40 antibody to elicit immunization against HPVE6/E7 successfully primed CD8 T cells to kill papillomavirus-related tumor cells *in vitro* [17]. Our data suggest that enhancement of effector function may be achieved by suppression of immune-inhibitory proteins.

## RESULTS

### E7 expression alters the kinetics of keratinocyte killing

**Figure 1:**
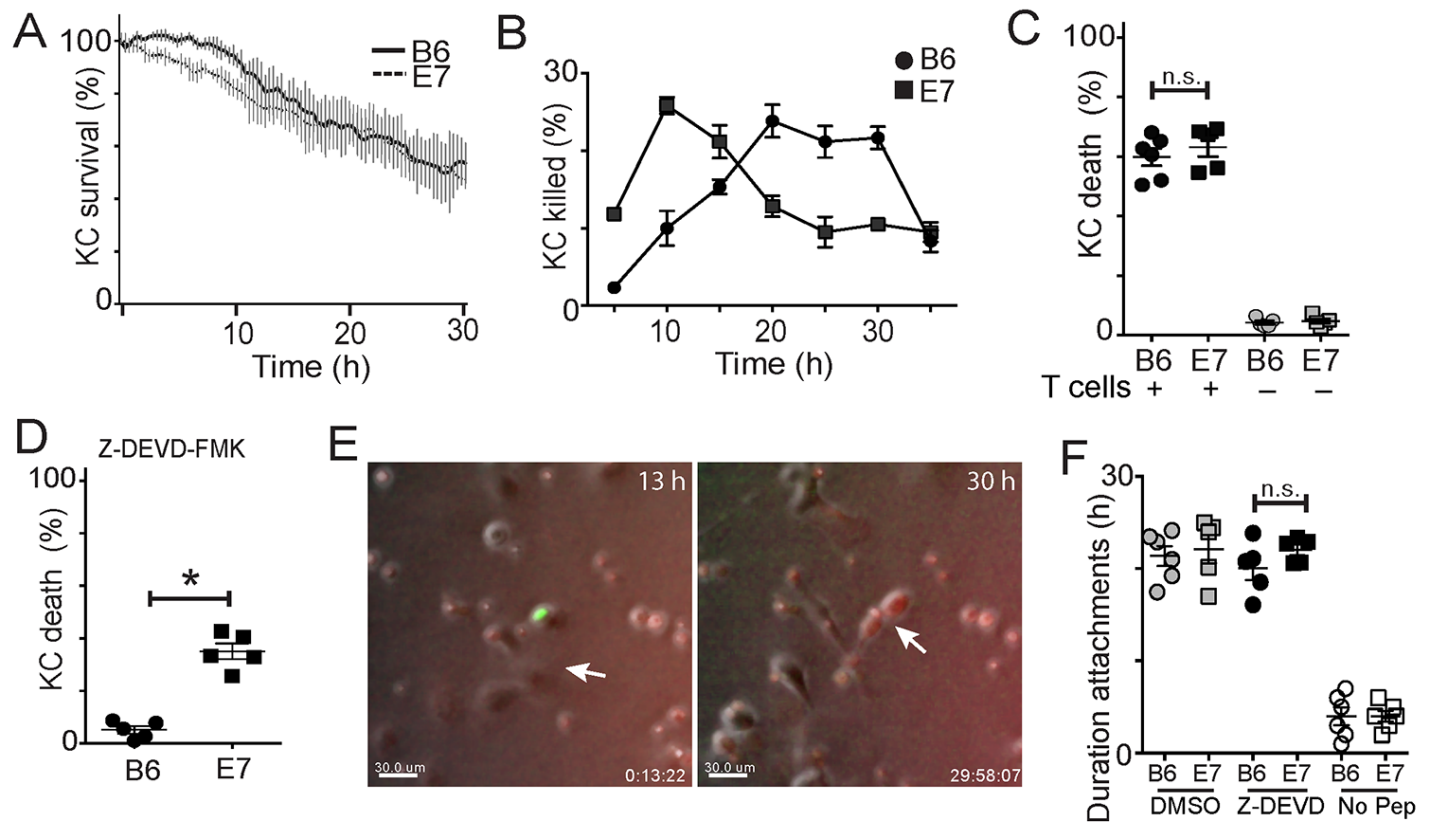
E7 expression by keratinocytes alters their susceptibility to killing by CTL Primary KC were isolated from B6 or E7 transgenic mice and loaded with SIINFEKL peptide. EGFP^+^OT-1 T cells were isolated and co-cultured with skin cells, with indicator dye for activated caspases. **(A)** KC survival over 30 hours of co-culture. Average of 4 experiments shown, error bars represent SD. **(B)** The percentage of KC deaths at 5 hour intervals was determined by counting newly dead cells at each time point and expressing as a fraction of the total number of cells in each frame. **(C)** KC death at 30 hours in co-culture with effector cells (black) or in monoculture (grey). **(D)** KC were incubated with Z-DEVD-FMK or DMSO (Mock) 60 minutes before and during co-culture; death assessed at 30 h. **(E)** CTL and KC co-cultures at 13 h showing attachment of CTL (green) to KC (arrow), and at 30 h showing early apoptosis of KC as indicated by red color change. Bar is 10 μm. See also, Supplementary Video 1. **(F)** Duration of attachments of E7-expressing (E7) or non-transgenic (B6) KC with CTL while incubated with DMSO (mock), Z-DEVD-FMK, or without peptide loading. (^*^p<0.05; n.s. not significant).

We investigated the effects of expression of HPV E7 oncoprotein by primary keratinocytes (KC) on their susceptibility to killing by CD8 T cells. K14.E7 mice (E7), derived from C57/B6 mice (B6), express HPV E7, a major oncoprotein in HPV-related cervical cancer, from the keratin-14 promoter. Thus HPV E7 is expressed in these mice predominantly by keratinocytes. We isolated primary keratinocytes from E7 mice, or from B6 mice, loaded them with SIINFEKL peptide, the TCR epitope of OVA, and co-cultured with CD8 OT-I T cells, which have a TCR receptor specific for SIINFEKL presented by H-2^b^. We found the total CTL-mediated killing of E7-expressing and non-transgenic KC to be the same over 30 hours (Figure 1A), which was consistent with other studies [19]. However, examining the kinetics of killing, B6KC exhibited specific “lag” period before target cell death (Figure 1A) which we have seen previously [18], while E7-expressing KC did not exhibit any lag period before death (Figure 1A), implying these cells may have altered killing kinetics. When loaded with the same dose of cognate peptide antigen, E7KC were killed earlier than non-transgenic cells (Figure 1B). The rate of KC death in monocultures and in co-cultures without peptide was similar between E7KC and B6KC, less than 7% over 30 hours (Figure 1C), showing E7 expression does not confer longevity on KC in culture. These data indicate that E7-expressing KC remain susceptible remain susceptible to killing by antigen-specific CD8 T cells, but possibly by different mechanisms to non-transgenic KC.

### Apoptosis of E7-expressing KC can follow a caspase-3 independent pathway

Both granule-mediated killing and Fas-mediated killing, the two primary contact dependent mechanisms used by CTL to kill their targets, predominantly involve activation of intracellular caspases, leading to activation of caspase 3 and resulting in cell death [20]. We investigated whether E7 expression altered the susceptibility of KC to be killed by caspase dependent mechanisms. Co-cultures of KC and CTL in the presence of FLIVO-SR dye that fluoresces red upon activation of intracellular caspases were treated with Z-DEVD-FMK, a specific inhibitor of caspase-3. Non-transgenic KC showed no progression to apoptosis as indicated either by cell morphology or by color change (Figure 1D, Supplementary Video 1). E7KC also had a marked reduction in the rate of apoptosis. However, around 35% of E7KC became apoptotic by color change and by morphology (p<0.05) (Figure 1D, E). Inhibition of caspase-3 did not affect duration of target-CTL attachment, which was similar in both E7 and B6KC in the presence of the caspase inhibitor (Figure 1F). We conclude that E7KC, but not B6KC, are susceptible to apoptosis by caspase-3 independent mechanisms.

### E7-expressing KC can be killed by perforin-independent mechanisms

**Figure 2:**
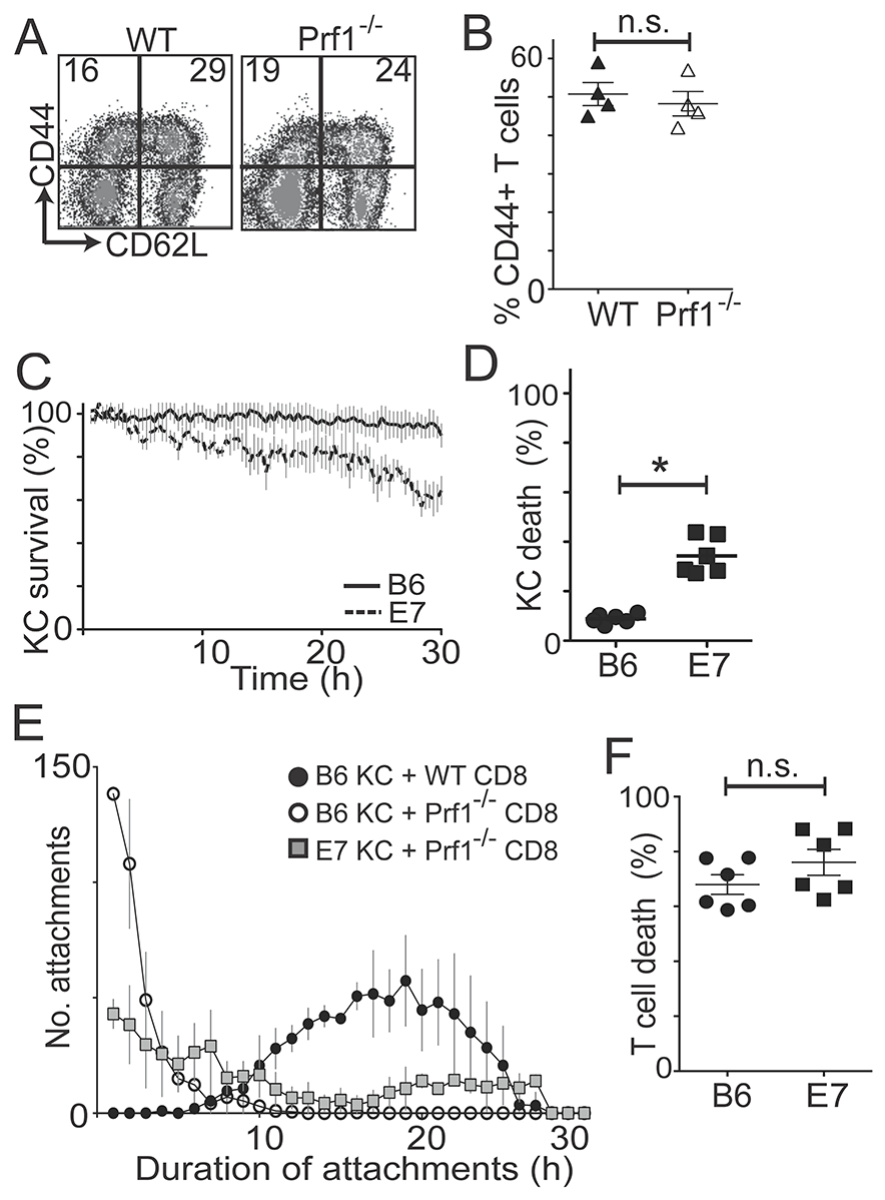
CD8 T cells kill E7KC by perforin-independent mechanisms *In vivo* and *in vitro* activated OVA-specific EGFP^+^Prf-competent and EGFP^+^Prf1^-/-^ CD8 T cells were added to KC primary cultures. **(A)** Flow cytometry of wild-type (WT) and perforin-deficient (Prf1^-/-^) CD8 T cells pre-gated on size and granularity, and CD8 expression. **(B)** Percentage of CD44^+^ cells after gating. Results of 4 independent CD8 harvests. **(C)** Primary KC from E7 and B6 mice were loaded with SIINFEKL and co-cultured with GFP^+^Prf1^-/-^ CD8 T cells. KC survival calculated as in Figure 1. **(D)** The final frames of movies were used to calculate percentage KC death over 30 hours. **(E)** Frequency distribution of the duration of attachments of effector cells to targets at 30-minute intervals. **(F)** Quantification of T cell death in co-cultures with antigen-loaded E7-expressing or non-transgenic KC at 30 hours. (^*^p<0.05; n.s. not significant).

Antigen-specific killing by CD8 effector cells classically occurs by granule-mediated mechanisms, which involve perforin-dependent delivery of granzymes to the target cell following direct CTL-target cell contact. To determine whether perforin is important in mediating KC killing by CD8 T cells, we immunized EGFP Prf1^-/-^ or EGFP B6 mice with SIINFEKL and isolated CD8 T cells, further culturing them with SIINFEKL peptide for three days to generate perforin-deficient or perforin-competent antigen-specific EGFP^+^CD8^+^ CTL of comparable activation status as assessed by CD44 and CD62L expression (Figure 2A, B). These effector cells were co-cultured with SIINFEKL-loaded E7KC or B6KC to assess target cell killing. In the absence of perforin, killing of non-transgenic KC was abrogated to baseline levels, while E7KC targets remained partially susceptible to CTL-mediated killing suggesting perforin was not necessary for killing of E7-expressing KC (p=0.02) (Figure 2C, D).

There was a marked reduction in the duration of attachment of effector cells to KC targets in the absence of perforin regardless of E7 expression (Figure 2E). Perforin-deficient CTL made numerous short-duration attachments to KC targets, lasting less than 60 min, before detaching without causing target cell death. However, when perforin-deficient CTL killed E7-expressing KC, they did so after an average attachment time of 18.4 h (± 4.2), compared with 13.3 h (± 3.6) for perforin-competent CTL (p=0.04), suggesting attachment time is relevant to the mechanism of target cell killing. Expression of E7 by KC did not affect T cell death rates (Figure 2F).

### E7KC are killed by TNFα-dependent and IFNγ-independent mechanisms

**Figure 3:**
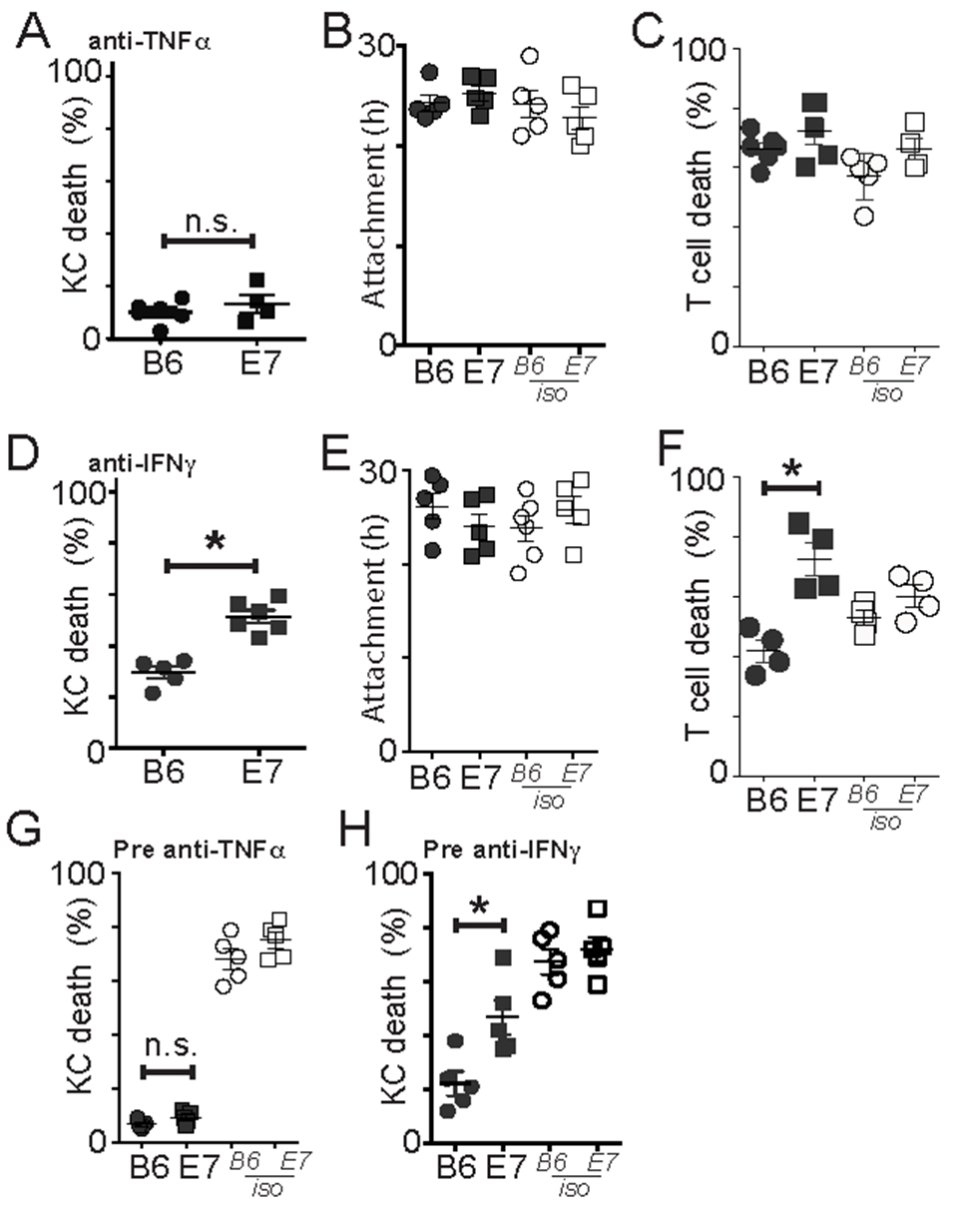
Perforin-competent CD8 effector T cells require TNFα but not IFNγ for cytotoxic function EGFP^+^OT-1 T cells were incubated with KC loaded with SIINFEKL. **(A-C)** An inhibitory antibody to TNFα was added to the KC before and during co-culture. KC death (A), duration of attachment of effectors to targets (B) and death of effector cells (C) with anti-TNFα or isotype control antibody. **(D-F)** Anti-IFNγ antibody was added to KC before and during co-culture. (D) KC death, duration of attachment (E) of effectors to targets and effector cell death (F) with anti-IFNγ or isotype control antibody. **(G-H)** Effector cells were pre-treated with antibody against TNFα (E) or IFNγ (F) before co-culture with target KC. Averages of 5 imaging sites of duplicate wells are shown as a single dot. Each dot is one separate experiment. Error bars are SEM. (^*^p<0.05).

Activated CD8 T cells produce TNFα and IFNγ that contribute to their cytotoxic function. We inhibited TNFα by incubating co-cultures of target and antigen-loaded effector cells with anti-TNFα inhibitory antibody. Anti-TNFα antibody completely abrogated killing of both E7-expressing and non-transgenic KC (Figure 3A), without affecting duration of cell-to-cell attachment (Figure 3B) implying CD8 T cells depend on TNFα to kill target KC. Inhibition of TNFα had no effect on death of T cells in the co-culture (Figure 3C). Inhibition of IFNγ, on the other hand, inhibited killing of B6KC, and partially inhibited killing of E7KC (Figure 3D) (B6 cf. E7 p<0.001; E7 isotype cf. anti-IFNγ p=0.016), also without affecting duration of effector cells attachment to targets (Figure 3E). Inhibition of IFNγ inhibited T cell death also in co-culture with B6KC, but not with E7KC, attributable to reduced antigen-induced cell death in the B6KC–T cell co-cultures (Figure 3F).

To confirm the inhibitory antibodies were acting on the CTL, rather than on the target cells, we pre-incubated the CTL with inhibitory antibody for 4 hours prior to co-culture with effector T cells. Inhibition of TNFα on CTL alone inhibited killing to a similar degree to treatment of co-cultures (Figure 3G). Inhibition of IFNγ on CTL, while it markedly inhibited killing of B6 cells, only partially inhibited killing of E7-expressing KC (Figure 3H), suggesting that IFNγ was not sufficient or necessary for CTL killing of E7-expressing KC.

### E7 expression alters KC susceptibility to CD8 T cell killing by Fas/FasL

Fas-FasL interaction is a major mechanism of inter-lymphocyte fratricide and CTL can express both Fas and FasL on their surface. Expression of Fas by tissue cells can be bound by CTL-related soluble or membrane-bound FasL resulting in target cell apoptosis, and may be an important mechanism of killing of tumor cells [21]. It is not known how E7 expression by keratinocytes affects their susceptibility to killing by the Fas/FasL system.

**Figure 4:**
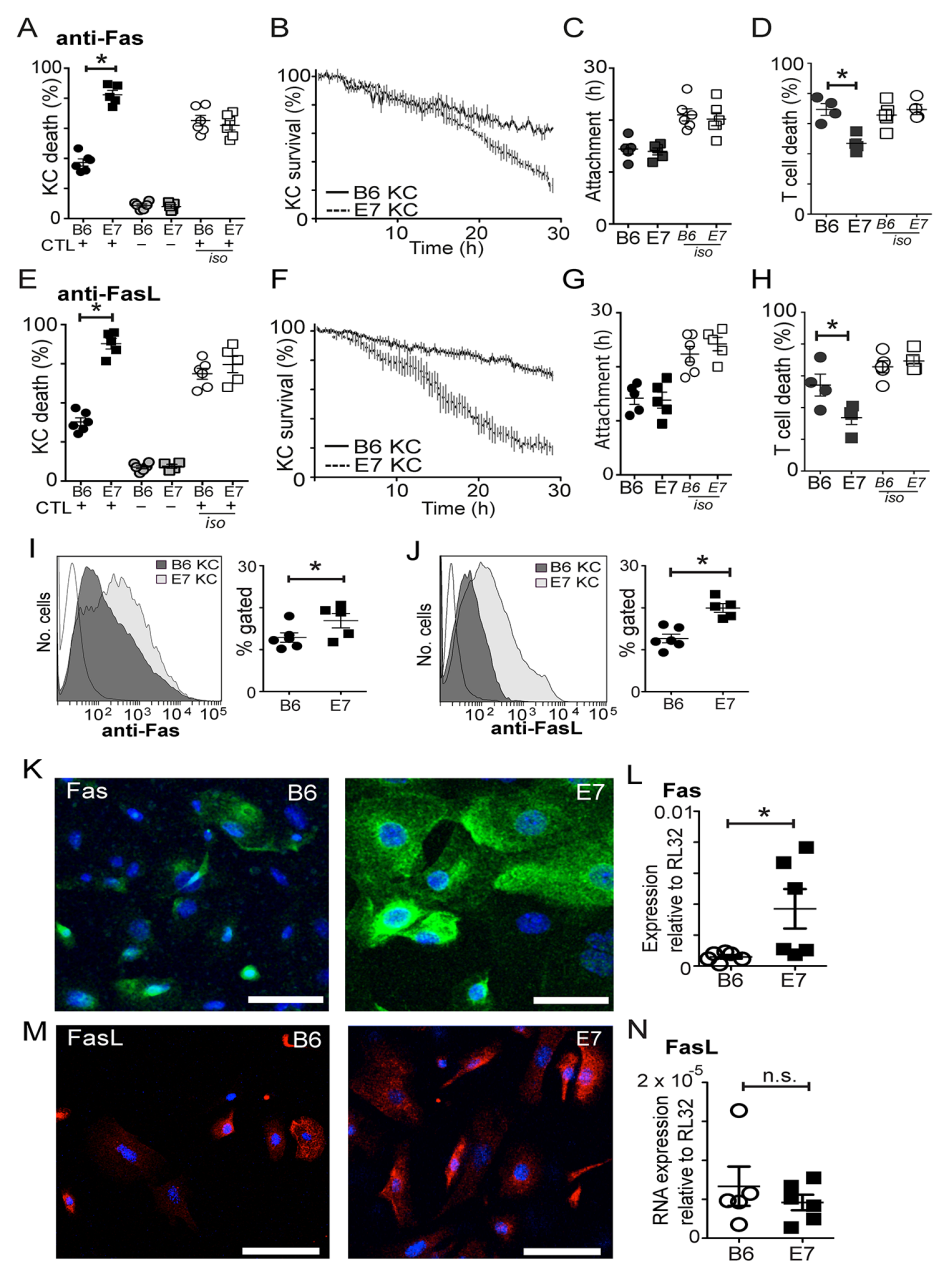
CD8 T cells utilize Fas/FasL to kill E7 expressing KC Co-cultures of effector cells and SIINFEKL-loaded KC were treated with inhibitory antibody to Fas **(A-D)** or FasL **(E-H)** or isotype control antibodies (iso). (A, E) Death of KC in co-culture (black) or monoculture controls (grey). (B, F) Survival kinetics of KC-effector co-cultures. (C, G) Duration of effector-KC attachment. (D, H) T cell death rate. Freshly-derived primary KC were examined for Fas **(I)** or FasL **(J)** expression by flow cytometry. Gating delineated living cells that were CD45 negative. Each value represents one mouse. **(K, M)** Confocal micrograph (maximal intensity projection) of primary KC cultured for 5 days and stained for surface expression of Fas (green) (K) or FasL (red) (M). **(L, N)** Primary KC were cultured for 5 days and cell lysates were harvested for RNA extraction. RT-PCR for Fas (L**)** or FasL (N) mRNA was performed comparing expression to a house-keeping gene. Each value represents one mouse. (^*^p<0.05; n.s not significant; bar is 50μm).

We incubated KC-CTL co-cultures with inhibitory antibody to Fas, or to Fas-L, both of which resulted in a significant, but not complete, inhibition of death of B6KC (Figure 4A and 4E; p=0.046 anti-Fas c.f. Isotype, p=0.049 anti-FasL c.f. Isotype). Inhibition of Fas and FasL also appeared to abolish the characteristic lag period before killing in these cells (Figure 4B, F). Inhibition of Fas or FasL reduced the duration of cell-to-cell attachment of both wild type and E7 transgenic KC (Figure 4C, G). Paradoxically, inhibition of either Fas or FasL caused a significant increase in killing of E7-expressing KC (Figure 4A, E; p<0.05 anti-Fas or anti-FasL c.f. Isotype). As CTL also express Fas, we examined the death rate of CD8 T cells in the co-cultures. With inhibition of either Fas or FasL, CTL death rate was significantly lower in co-cultures with E7KC, but not B6KC (Figure 4D, H), suggesting increased availability of cytotoxic T cells may be the mechanism by which Fas/FasL inhibition enhanced E7KC killing in this assay.

Therefore we examined E7KC and B6KC to determine whether E7 expression increased the expression of Fas or FasL on the surface of the cells. Flow cytometry of freshly harvested KC showed increased Fas and FasL expression on the surface of E7KC compared with wild type KC (Figure 4I, J). Confocal microscopy after five days in culture allowing protein expression and cell division to recommence, showed that, although variability in expression levels between cells in the same culture was evident as expected with cycling cells, there was a marked increase in surface expression of Fas and FasL in E7-transgenic KC (Figure 4K, M). Examination of the RNA expression of cultured KC confirmed up-regulation of Fas mRNA expression in E7KC compared with wild type cells (Figure 4L), although FasL mRNA expression was not up regulated suggesting, as have others, that its regulation is post-transcriptional [22] (Figure 4N).

These data allow the hypothesis that E7KC are more susceptible to apoptosis via Fas on their cell surface, and that increased expression of FasL may allow E7KC to kill effector T cells.

### PD-L1 expression by E7-expressing KC is protective

**Figure 5:**
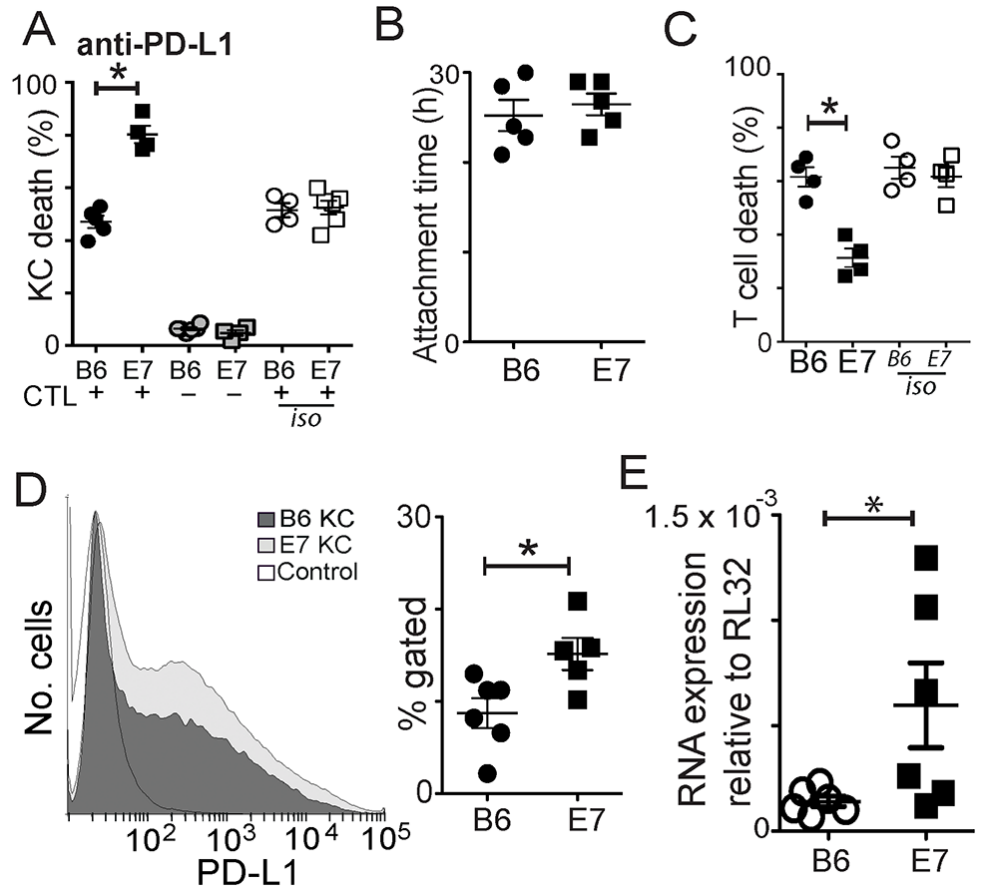
Increased levels of PD-L1 expression by E7KC is protective Co-cultures of CTL and SIINFEKL-loaded B6KC or E7KC were treated with anti-PD-L1 antibody or isotype control antibody. **(A)** Death of KC in co-culture with effectors (black) or as monocultures (grey), or in co-cultures without PD-L1 inhibition (white). **(B)** CTL-target attachment duration. **(C)** CTL death in co-cultures. **(D)** Flow cytometry of primary KC harvested as in Figure 4E, and stained for PD-L1 expression using the same gating strategy. Isotype control is also shown. **(E)** Primary KC were cultured for 5 days and cell lysates were harvested for RNA extraction and subject to RT-PCR for PD-L1 mRNA quantification. Each value is one mouse. (^*^p<0.05).

Epithelial cell expression of PD-L1 as with FasL expression, has been associated with resistance of tumors to T cell mediated killing. PD-L1 is expressed on many epithelial cells, usually affording immune protection, as interaction with PD-1 on activated T cells leads to down regulation of T cell function. We found that inhibition of PD-L1 in co-cultures of effector and target cells had little effect on killing of non-transgenic KC (Figure 5A; p=0.15 untreated c.f. anti-PD-L1 in B6KC), but promoted killing of KC expressing E7, suggesting up regulation of PD-L1 as a result of E7 expression contributes to resistance to CD8 T cell killing (Figure 5A; p=0.016 E7 c.f. B6; p=0.03 untreated c.f. anti-PD-L1 in E7KC). Unlike FasL inhibition, PD-L1 inhibition did not affect the duration of CTL/target cell attachment (Figure 5B). As the reduced killing may have been the result of an increasing ratio of effectors to target cells as the culture progressed, we investigated whether T cells were failing to die in these treated co-cultures. Inhibition of PD-L1 markedly reduced the death rate of CTL in E7KC co-cultures, implying that some of the CD8 T cell death was attributable to PD-L1 expressed on the surface of transgenic KC (Figure 5C).

We investigated whether E7 expression on KC altered their expression of PD-L1. Freshly isolated E7-transgenic KCs were subject to surface staining for PD-L1. We saw a significant increase in the surface expression of PD-L1 by flow cytometry in these cells (Figure 5D). RT-PCR studies of cultured KC at 7 days showed marked increase in PD-L1 RNA expression among E7KC compared with non-transgenic cells (Figure 5E).

### Hierarchical killing mechanisms used by CTL to kill E7-expressing KC

CD8 T cells have been shown to utilize different mechanisms of cytotoxicity in a hierarchical, tissue-dependent manner [23]. In order to investigate how E7 oncoprotein expression affected the hierarchy of mechanisms used in killing KC, we now combined different combinations of the killing mechanisms recognized as being relevant to tumor killing to rank the importance of each. Not all combinations were included, particularly where the primary cytotoxic effect was so marked as to render any further effect immeasurable and irrelevant.

**Figure 6:**
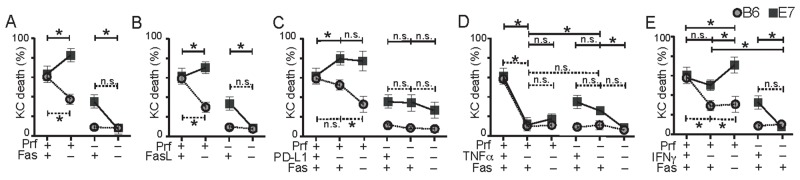
Hierarchical killing of KC by CTL is altered by E7 expression T cell killing mechanism hierarchy was investigated by inhibition of multiple pathways during co-culture of primary non-transgenic KC (B6) or E7-expressing KC (E7) loaded with constant amount of peptide and perforin-competent or Prf1^-/-^ effector CD8 T cells. Co-cultures were incubated with anti-Fas **(A)**, or anti-FasL **(B)** inhibitory antibody. **(C)** Co-cultures of effector and target cells were incubated with anti-PD-L1 inhibitory antibody, with or without Fas inhibition. **(D)** Co-cultures were treated with anti-TNFα antibody with or without Fas inhibition. **(E)** Co-cultures were treated with anti-IFNγ with, or without, Fas inhibition. Average values of five imaging sites of duplicate wells for each experiment are shown as one dot. (^*^p<0.05) Data from single inhibitor experiments Figure 2-5 have been reshown in these graphs for ease of comparison. Note that the ‘+’ indicates the functional presence of the particular cytokine while ‘–’ indicates its absence by knock-out or by antibody (e.g. If Fas is present, there is a ‘+’, while Fas inhibition with antibody is indicated by ‘–’).

We co-cultured primary B6 or E7-expressing KC loaded with SIINFEKL with OVA*-*primed effector cells from perforin-competent B6 mice or from perforin-deficient Prf1^-/-^ mice, and inhibited Fas or FasL. Inhibition of the Fas pathway abrogated the non-perforin mediated killing of E7-expressing KC seen in Figure 2 to levels similar to that of non-transgenic cells (Figure 6A, B). Fas had no protective effect on killing of E7KC in the absence of perforin.

Perforin-dependent B6KC killing, which was partially reduced by inhibition of PD-L1, was further reduced by inhibition of Fas. Similarly, the protective effect of PD-L1 on E7KC was not seen in the absence of perforin as inhibition of PD-L1 resulted in abrogation of perforin-independent killing in E7KC (Figure 6C).

Likewise, although TNFα inhibition of perforin-sufficient T cells abrogated KC death, in perforin-deficient co-cultures, the reduction in KC death was much less, implying that perforin-mediated killing was more dependent on TNFα than perforin-independent killing. Additional inhibition of Fas was not protective of E7KC death by perforin-sufficient CTL and did further impede killing by perforin-deficient CTL. (Figure 6D).

IFNγ has been shown to enhance cytotoxic killing of target cells by inducing antigen expression and increased cytokine production. In immune-mediated killing of E7-expressing KC, its role *in vivo* appears to be inhibitory [24]. *In vitro*, inhibition of IFNγ did not significantly affect CTL killing of E7KC (Figure 6E), but inhibiting Fas in this environment increased E7 target cell loss, suggesting the protective effect of Fas on E7 killing is independent of IFNγ. In the absence of perforin, on the other hand, inhibition of IFNγ abrogated killing. These data suggest IFNγ plays a role in the perforin-independent killing of E7KC.

**Figure 7:**
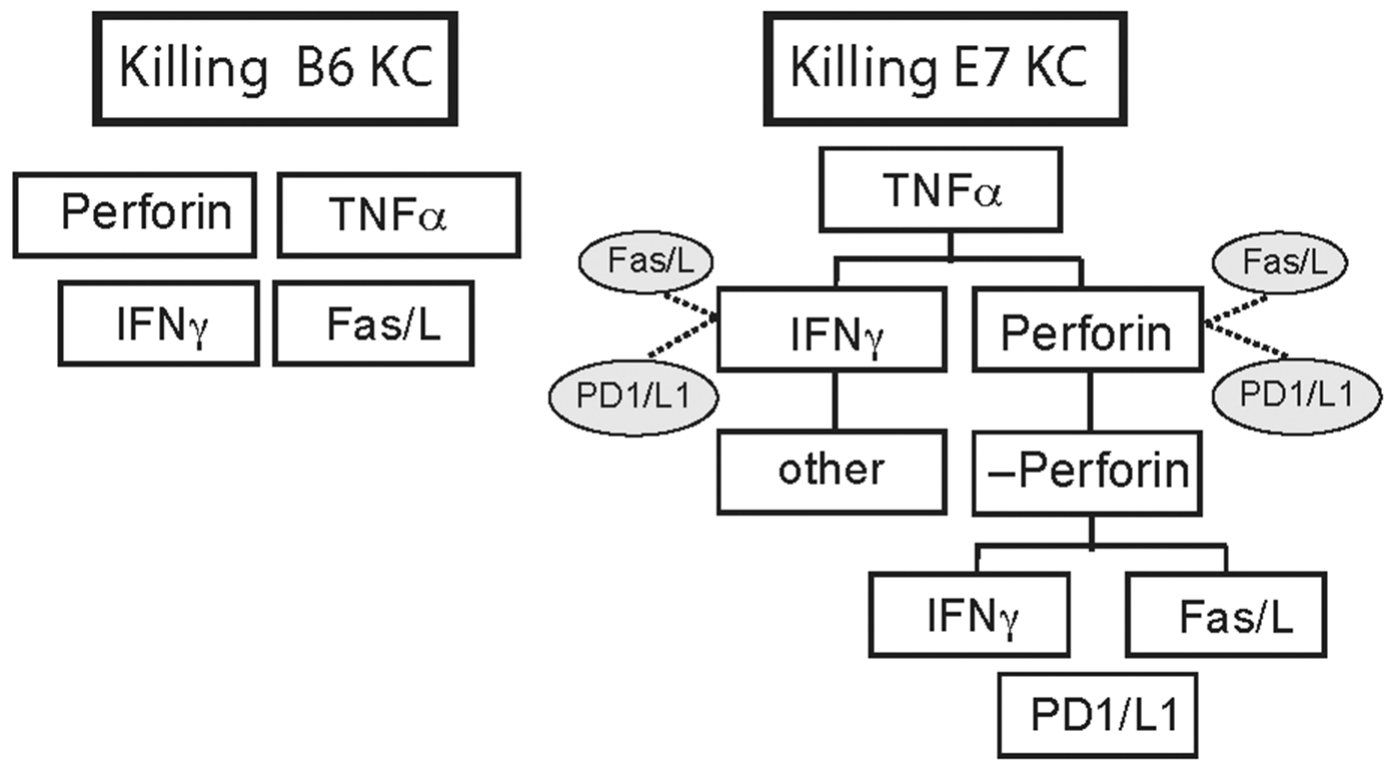
E7 expression by KC alters hierarchy of killing mechanisms used by CTL Putative differences in the hierarchy of killing mechanisms used by CTL to kill KC expressing E7 compared with non-transgenic B6KC targets as modeled by *in vitro* killing studies in this study. Cytokines inhibitory to a specific killing mode are indicated by dotted lines. Without oncogene expression, B6KC were killed predominantly by perforin and TNFα mediated mechanisms, both of which were essential, while Fas/FasL and IFNγ were less important modes of killing. PD-L1 is not expressed in B6KC, and blockade had no effect on killing, and this has been excluded from the diagram. With expression of E7 oncogene, E7KC still require TNFα as essential for killing. Less significantly, IFNγ and perforin contribute to killing targets. IFNγ-independent killing does take place, but it appears not to be mediated by any of the processes we examined here. Perforin-independent killing is reliant on Fas/FasL or on IFNγ. PD1/L1 contributes to target cell death perforin-independent killing, but along with Fas/FasL, inhibits killing by IFNγ and perforin in perforin-sufficient T cells.

Based on these tests, we ranked the mechanisms of killing used by CTL to achieve target cell death of B6 and E7KC. CTL killing of non-transgenic KC relies predominantly on perforin and appears to be licensed by TNFα. Further down the hierarchy, but playing an important role are IFNγ and the Fas/FasL system, while PD1/PD-L1 system plays no role in KC killing. TNFα plays a key role among the mechanisms CTL used to kill E7KC. Ranked below this, and equally significant *in vitro* are IFNγ and perforin, while the Fas/FasL system and PD1/PD-L1 system offer some protection for E7KC against these mechanisms. The percentage of killing of E7KC that is perforin-independent may be due to IFNγ or the Fas/FasL system, while TFNα plays only a minor role in this system (Figure 7).

## DISCUSSION

CD8 T cells, the primary effector cells in tumor eradication, utilize a variety of seemingly overlapping and redundant mechanisms to effect target cell death, both contact-dependent and contact independent, a trait particularly important to their anti-viral functions [25]. The relationships between these redundant mechanisms are highly complex and likely offer an evolutionary advantage. Here we show that these mechanisms can be used hierarchically in response to oncogene expression. This study does not examine the effect of priming and the response of the T cell to the specific antigen expressed by the target cell, rather it examines how expression of the oncogene alters protein expression within the cell to require effector T cells to utilize different mechanisms of killing in order to effect target cell death.

CD8 T cells are known to predominantly use perforin to kill target cells. Earlier studies identified TNFα-mediated killing mechanisms as equally significant in achieving target cell death, in both infectious and non-infectious models [26, 27], which we confirm here. We also found that with non-oncogene expressing KC, IFNγ and Fas/FasL were less critical to achieve target cell death, and supplemented the primary mechanisms. Fas and IFNγ have been reported to be more important in killing pancreatic beta cells in T-cell dependent murine models of diabetes mellitus [28] although TNFα assistance was essential [23], as was also confirmed in this model.

The factors that determine the choice of mechanism used by CD8 effector cells to achieve target cell death remain unclear. Target cell type likely plays an important part, as death is determined by the concentration of specific receptors on the target cell surface such as FasL, IFNγR and negative ligands such as Fas and PD-L1. Keratinocyte susceptibility to non-perforin mediated killing is markedly up-regulated in the absence of PD-1/PD-L1 interaction [29], suggesting that redundant killing mechanisms are preferentially up-scaled when primary mechanisms are not available. Additionally, the inflammatory environment and cytokine milieu may play a key role in determining the actions of infiltrating effector cells. Extreme inflammatory environments lead to large amounts of locally produced TNFα and IFNγ which, in addition to being cytotoxic to KC directly, also up-regulate Fas-mediated KC death [30, 31]. The strength of the signal to T cells has also been implicated in determining the mechanism of cell death, with high-affinity ligands leading to perforin-mediated killing while low affinity ligands utilize slower perforin-independent pathways [32]. Additionally, the use of perforin and Fas by CTL has been associated with strength of priming in antigen-specific killing and Fas expression may improve perforin-mediated killing [33, 34].

Pathogenic organisms, such as viruses, adapt their host cell to avoid being killed rapidly by perforin, which may leave them more susceptible to slower Fas/FasL interaction for eradication [35], thus giving the organism time to replicate and disseminate. In this study, utilization of Fas/FasL resulted in markedly increased contact time between effector and target cells. In the E7 transformed KC with its increased rate of replication, this may represent a mechanism for immune cells to escape killing by Fas. We speculate that the prolonged contact time required for Fas-mediated killing may promote modalities of cellular escape by target cells during this phase of attachment such as alteration of membrane dynamics during mitosis. The effects of target cell mitosis on immunological killing mechanisms is unknown and we suggest the utilization of imaging techniques is assessing effector-target contact in tumors is a useful in assessing this further. In addition, FasL expression on endothelial cells has been shown to delay T cell migration into tumours and may have an effect on T cell motility which we have previously shown is directly related to tumour killing ability by these cells [18, 36].

HPV E7 expression confers changes upon host keratinocytes that may have had a growth advantage for the virus which persist upon transformation. In immortalized cell lines and transformed KC, E7 expression confers resistance to the direct cytostatic effects of TNFα possibly by the up-regulation of cell cycling and replication due to the action of E7 on the retinoblastoma gene [10, 37]. We did not assess KC replication in our model, but suggest additionally that oncogenes such as E7 may alter the expression levels of surface markers reliant on TNFα stimulation, such as Fas and IFNγR, which are required for CD8 T cell mediated killing. This is also supported by evidence that the addition of TNFα to tumor cells increases their susceptibility to Fas-mediated killing by effector cells [38].

HPV-related cervical cancer and head and neck cancers are associated with increased expression of PD-L1, which is associated with dysfunction of the T cell immune response to these tumors [16, 39]. We show here that E7 expression on KC is sufficient to up-regulate of these negative receptors resulting in both KC protection and in increased death of effector cells, promoting a fight-back response. Tumors are associated with a infiltration of T cells displaying varying degrees of exhaustion [40], characterized by low levels of perforin and IFNγ expression, and increased expression of negative regulators such as PD1. Thus perforin-independent mechanisms of T cell killing become important in maintaining some degree of anti-tumor activity. Interestingly, IFNγ-independent killing of E7KC took place that is not dependent on any of the other methods of killing we have examined here, emphasizing that CD8 T cells have an extensive armamentarium of killing, and we have explored only some of the major ones in this study. Molecules such as Tim-3 have shown increasing promise as therapeutic targets and remain to be explored [41].

Understanding the changing nature of susceptibility to immune-modulated killing mechanisms due to oncogene expression holds the key to optimizing immunotherapy for HPV-related cancers. Using a protease inhibitor to sensitize tumors to apoptosis resulted in enhanced IFNγ production, increased Fas expression and improved tumor lysis *in vivo* [42]. Likewise, inhibition of PD1/L1 reduces T cell death and improves eradication of HPV-related tumors in immunomodulatory therapy [43]. Recently, an anti-CD40 based vaccine against E7 was able to prime T cells to kill HPV-related tumor cells, suggesting that once cytotoxic T cells are primed against E7 targets, their killing may be optimized to achieve strong tumorcidal efficacy [17]. Additionally, chemotherapeutic agents may be immunologic sensitizers to immunotherapy, modulating cancer targets for better recognition and killing [44]. Optimization measures could include a combination of blockade of negative regulators Fas/FasL and PD1/L1, along with cell sensitization therapies to improve IFNγ and perforin-mediated killing. Further investigation of IFNγ-independent killing of E7-expressing KC may lead to more therapeutic targets.

In this study, we employed a minimalist model *in vitro* using primary murine cells to examine the relative importance of several redundant killing mechanisms used by cytotoxic T cells in effecting tumour cell death. These mechanisms may reflect only part of the interactions *in vivo* in human disease in which levels of inflammatory cytokines and variable expression of cognate antigen play important roles.

## MATERIALS AND METHODS

### Mice

All experiments were approved by the University of Queensland animal ethics committee. K14.E7 mice from a B6 background were bred in-house. Rag1^-/-^ mice were purchased from the Animal Resource Centre, Perth, Australia. B6.Nzeg mice express a nuclear-localised enhanced green fluorescent protein (EGFP) and were provided by K. Matthaei, Australian National University. B6.Prf1^-/-^ mice were bred with B6.Nzeg mice to generate Prf1^-/-^. Nzeg mice, also expressing EGFP.

### Reagents

Anti-CD44, anti-IFNγ, anti-CD8, relevant isotype antibodies as well as inhibitory antibodies to Fas, FasL, TNFα, and INFγ were purchased from BD Pharmingen. Anti-CD62L was from eBioscience. Recombinant IFNγ was purchased from R&D Systems. Z-DVED-FMK and ovalbumin (OVA) were purchased from Sigma. Media for cell culture and IL-2 was purchased from Invitrogen.

### Keratinocyte culture

Primary KC were harvested from 3-4 day old E7 and B6 mice as described [13]. Keratinocytes were cultured in 24-well culture plates (allocating 12 wells to each KC type) in DMEM with 5% fetal bovine serum for 24 hours before changing to Serum-Free Media supplemented with bovine pituitary extract and epidermal growth factor. The 24-well plate of KC was imaged when the cells were 70% confluent.

### Harvest of CD8 T cells

EGFP^**+**^ mice were immunised by subcutaneous injection with 50 μg of OVA, and Quil A (20 μg). Splenocytes were harvested one week later, and stained with anti-CD8, before FACS to obtain a >98% pure population of GFP^+^CD8^+^ cells. These were further cultured for three days in the SIINFEKL peptide, the minimal CTL epitope of OVA, and IL-2 to further select for OVA-specific CD8 T cells.

### Co-culture

C57 and E7KC were loaded with 1 μg.ml^-1^ of SIINFEKL peptide for 60 min at 37°C once they reached 70% confluence in culture on 24-well plates. KC were extensively washed in warm PBS before co-culturing with effector cells. We added 5 ×10^3^ EGFP^+^CD8^+^ T cellsper well into duplicate wells of keratinocytes, giving an approximate target:effector ratio of 10:1. Cells were imaged at 37°C with 5% CO_2_ for 30 h in 50% serum-free medium, 45% RPMI, 5% fetal bovine serum, 5 ng. ml^−1^ IL-2 and 2% SR-FLIVO solution. We included control wells of KC monocultures in each plate, as well as co-cultures without peptide loading, to assess baseline KC death and ensure there was no non-antigen specific killing as previously described [18]. Inhibitory antibodies or isotype control antibodies were added to the KC at the time of CTL addition unless stated otherwise. In pre-incubation experiments, KC were incubated with antibodies or isotype controls for 60 min and cells were then washed four times with media prior to the addition of CTL.

### Cell killing assay

As previously, activation of intracellular caspases was noted by a color change in the cells mediated by cell-permeant SR-FLIVO red (courtesy of Immunochemistry Laboratories), as determined by image analysis software [18]. Rates of KC death of cells were determined by size (>12 μm for KC and <10 μm for T cells), and expressed as a percentage of number of cells of that type visible at time 0. We used at least 3 fields of view per well of duplicate wells from at least 3 replicated experiments.

### RNA analysis

Ear skin samples were lysed into RNase-free microtubes in 1ml of Trizol (Sigma) and RNA extracted according to manufacture’s instructions. The RNA pellet was washed twice in 70% ethanol and air-dried before resuspension and genomic DNA was then digested as per manufacturer (Qiagen). RNAs were quantified by nanodrop spectrophotometry. For retro-transcription, 500 ng of RNA combined with 25 mM MgCl_2_, 25 mM dNTPs, oligoDT, RNase inhibitor, and MuLV Taq polymerase in its buffer for 25 min at 25°C, 60 min at 42°C and 5 min at 95°C. Samples were amplified using Sybr premix Taq II (TAKARA) on ABI7900 (Applied Biosystems) - 1x 30 s at 95°C, 45x (5s 95°C and 30 s at 60°C), followed by (15 s at 95°C, 60 s at 60°C, 15 s at 95°C). Primers were designed using Integrated DNA Technologies.

### Immunofluorescence

Cells for flow cytometry were prepared by trypsination of co-cultures, followed by viability staining, and then surface staining with anti-Fas or anti-FasL antibody or isotype-specific negative controls at 4°C before running on BD FacsCanto with Diva software. Semi-quantitative analysis was determined only as a percentage of total cells by flow cytometry. For microscopy studies, co-cultures were performed on chamber slides, which were then harvested, fixed in 1% paraformaldehyde and stained for surface antigen expression.

### Microscopy

Confocal microscopy was performed on a Zeiss Meta-510 confocal microscope using Zen software, with ×25 oil-immersion objective lens. Time-lapse microscopy was performed on a Zeiss Axiovert 200M microscope with a ×10 objective lens. Images were acquired from five fields of view per well from duplicate wells every 12 min for 30 h. Cells were maintained with humidification, in 5% CO_2_, at 37°C during imaging by specialized enclosure built around the microscope.

### Image analysis

Imaris software was used to de-convolve and analyze raw images, and generate movies. Death of KC and T cells was determined by caspase-3 activation and resultant red color change. Statistical analysis was performed using Excel and Graphpad Prism. To prepare images for publication, cropping, a median filter (3×3), and minor contrast adjustments were made using Adobe Photoshop or Imaris.

### Statistics

Data were collected from 5 sites each from duplicate wells and averaged giving a single value for each experiment, and experiments were repeated at least 3 times. We used the one-tailed Mann-Whitney test, or one-way ANOVA to compare non-parametric data. We accepted p < 0.05 as showing significant difference between groups.

## SUPPLEMENTARY MATERIALS VIDEO





## Abbreviations

HPV: human papillomavirus
KC: keratinocyte
OVA: ovalbumin
EGFP: enhanced green fluorescent protein
