# Supplementary material for: Human papillomavirus E7 oncoprotein expression by keratinocytes alters the cytotoxic mechanisms used by CD8 T cells
Source: Oncotarget. 2017 Dec 14;9(5):6015–27. doi: 10.18632/oncotarget.23210 (PMC5814191; doi:10.18632/oncotarget.23210)
Supplement: Supplementary file 1 [file oncotarget-09-6015-s001.pdf]

Human papillomavirus E7 oncoprotein expression by keratinocytes alters the cytotoxic mechanisms used by CD8 T cells

SUPPLEMENTARY MATERIALS

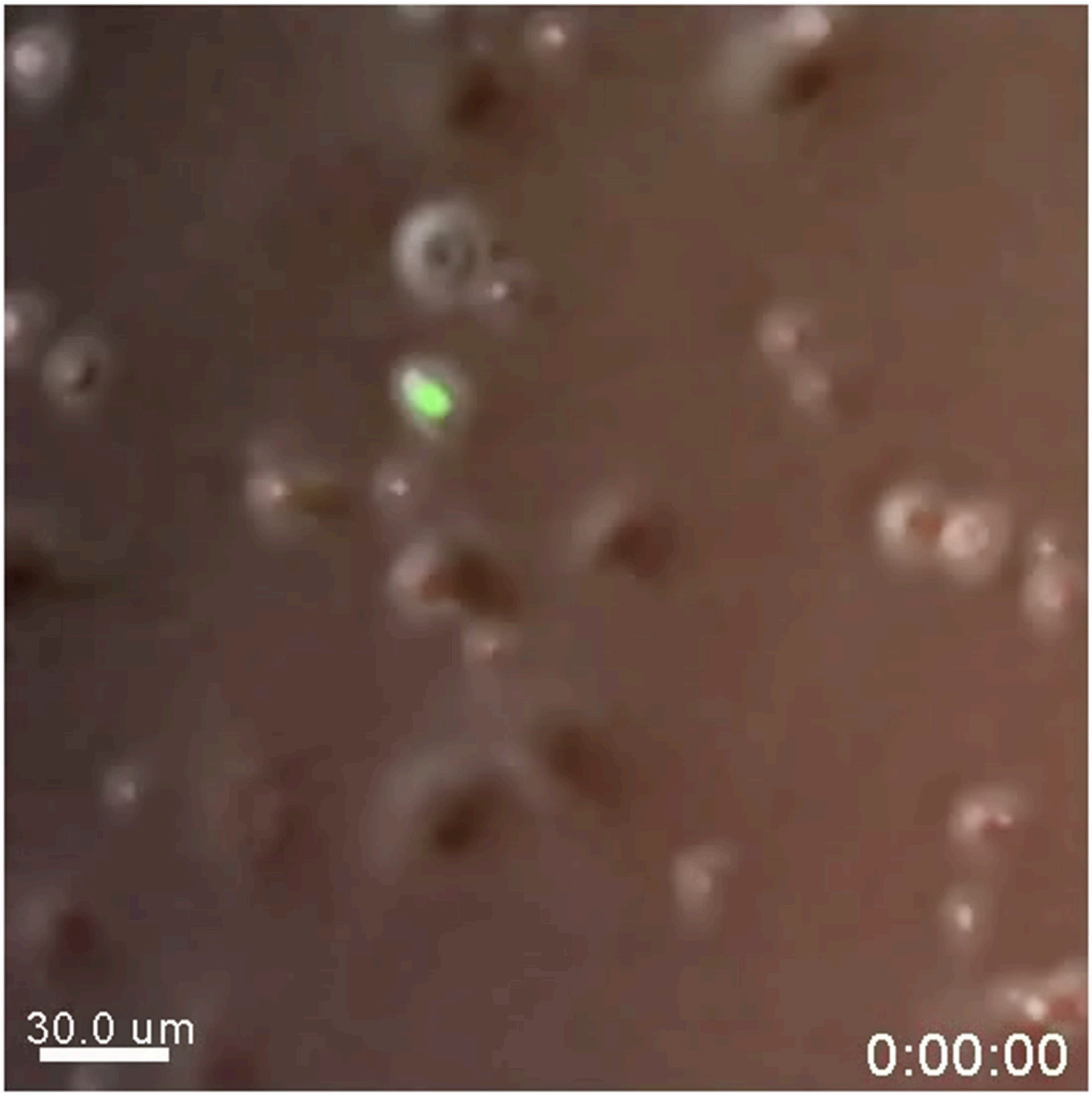

Supplementary Video 1: EGFP$^+$CD8$^+$ T cells kill KC targets loaded with cognate peptide. Example of extended-duration microscopy of KC (larger unstained cells) and EGFP$^+$ effector T cells. Imaging is over 30 hours. The highly motile T cell (green) makes contact with KC targets within 30 min and remains attached for around 5 hours before detaching and moving away. The target cells die after contact several hours later becoming positive for caspase-3 at around 16 hours as indicated by intracellular increase in concentration of indicator dye (red). The T cell also subsequently dies, becoming red. Several other caspase-3 positive and negative KC are visible in the field of view.

See Supplementary Video 1
